# Immunological response and overall survival in a subset of advanced renal cell carcinoma patients from a randomized phase 2/3 study of naptumomab estafenatox plus IFN-α versus IFN-α

**DOI:** 10.18632/oncotarget.2922

**Published:** 2015-02-14

**Authors:** Eyad Elkord, Deborah J. Burt, Anette Sundstedt, Örjan Nordle, Gunnar Hedlund, Robert E. Hawkins

**Affiliations:** ^1^ Department of Medical Oncology, Institute of Cancer Sciences, The University of Manchester, Manchester, UK; ^2^ Department of Medical Microbiology & Immunology, College of Medicine & Health Sciences, United Arab Emirates University, Al Ain, UAE; ^3^ Biomedical Research Centre, School of Environment & Life Sciences, University of Salford, Salford, UK; ^4^ Active Biotech AB, Lund, Sweden

**Keywords:** Renal cell carcinoma, naptumomab estafenatox, ABR-217620, immune analysis, overall survival

## Abstract

Naptumomab estafenatox/ABR-217620/ANYARA (Nap) has been evaluated in clinical phase 1 and 2/3 studies. RCC patients in the phase 2/3 trial were randomized 1:1 in an open label study to receive Nap+IFN-α or IFN-α. In this study, we analyzed the UK patients for their immunological response in relation to prolonged overall survival (OS). We found that Nap-specific T cells were reduced after 3 treatment days in patients' peripheral blood. Levels of both Nap-specific CD4^+^ and CD8^+^ T cells were significantly higher 8 days after the first treatment. Patients with such pattern of reduction and expansion of Nap-binding T cells also showed increased levels of IL-2 and IFN-γ in plasma 3 hours after the first Nap treatment. In addition, Nap caused an increase of IL-6, IL-10 and TNF-α. The patients in the UK subset showed a tendency of OS benefit after Nap treatment. Most Nap treated patients with long OS had low baseline IL-6 and normal levels of anti-SEA/E-120 antibodies. Furthermore, patients with pronounced Nap induced IL-2 and T cell expansion had long OS. In conclusion, patients with low baseline IL-6 and normal anti-SEA/E-120 may respond well to Nap by T cell activation and expansion paving the way for anti-tumour effects.

## INTRODUCTION

Renal cell carcinoma (RCC) accounts for 2–3% of all new cancer cases [1]. The clear cell RCC is the most common subtype and accounts for 70–80% of all renal cancers. Although RCC is resistant to classic chemotherapy, development of new therapies including tyrosine kinase inhibitors has improved the median survival period of patients with advanced RCC to about 26 months. Improvement of therapy of RCC by introducing new concepts is still urgent though [1]. Immunotherapy is well on the way to becoming an established tool in the cancer treatment armory and RCC is regarded as a sensitive tumour type.

Antibody targeting of superantigens to tumour cells combines powerful T cell activation and cytotoxicity with a targeted approach to eradicate tumour cells [2]. Tumour targeted superantigens (TTS) are recombinant fusion proteins that consist of an anti-tumour Fab moiety genetically fused to a superantigen [3, 4]. TTS treat tumours via the local activation of the patient's cytotoxic T cells through a process termed Superantigen Antibody Dependent Cellular Cytotoxicity (SADCC) [5]. Using this approach, 5T4 tumour-associated antigen was targeted by genetically fusing murine Fab fragment of the monoclonal antibody 5T4 to a mutated superantigen staphylococcal enterotoxin A (SEA), and maximum tolerated dose and safety established in a trial in non-small cell lung carcinoma (NSCLC) patients [6]. A phase II study of RCC patients receiving at least two four-day cycles of doses of anatumomab mafenatox (ABR-214936) treatment approximately one month apart was completed. When stratified by the Memorial Sloan-Kettering Cancer Centre (MSKCC) prognostic criteria, a prolonged survival compared to published expectation was observed. The group of patients receiving the highest drug exposure lived almost twice as long as expected, while the low dose group lived as expected from their MSKCC risk scores [7]. The increase in circulating interleukin (IL)-2 levels after treatment provides a useful biomarker for clinical effect since patients with the highest increase in IL-2 at the second treatment day lived significantly longer [7]. The next generation of drug, Naptumomab estafenatox/ABR-217620/ANYARA (Nap), was then developed. Nap, as anatumomab mafenatox, contains the 5T4 antibody but uses the mutated SEE SEA/E-120 superantigen [8]. The 5T4 antibody recognizes an oncofoetal antigen, a transmembrane glycoprotein which is expressed by different cancers; it is expressed at high levels in 95% of RCC [9]. Restricted expression of 5T4 on tumour tissues as well as its association with tumour progression and poor prognosis has driven the development of 5T4 vaccine- and antibody targeted immunotherapies [10]. The fusion protein 5T4 antibody moiety has an affinity in the order of 1 nM. Nap induces T cell mediated killing of tumour cells, SADCC, at concentrations around 10 pM and the SEA/E-120 superantigen moiety has been engineered to have low binding to human antibodies and MHC class II [8, 11].

After phase 1 studies [12] a prospective, randomized phase 2/3 trial was conducted. Patients with RCC were randomized 1:1 in an open label study to receive Nap+IFN-α or IFN-α: 15 μg/kg Nap was given intravenously in three cycles of four once daily injections plus IFN-α (9 MU subcutaneously three times weekly) or the same dose and schedule of IFN-α monotherapy. We have previously presented the final results (ASCO annual meeting 2013 abstract ID 3073, European Cancer Congress (ECCO) 2013 abstract ID 2710 and manuscript submitted for publication). Although the study did not meet the primary endpoint, addition of Nap to IFN-α might improve OS and PFS in a subgroup of patients with low IL-6 and normal levels of anti-SEA/E-120 antibodies. Furthermore it was shown that patients from certain territories had increased levels of baseline anti-SEA/E-120 and low Nap exposure. Patients in the United Kingdom (UK) had expected levels of anti-SEA/E-120 and Nap exposure. During the study in the UK, we extended sampling in association to Nap treatment to include also peripheral blood mononuclear cells and analyzed Nap induced changes in different lymphocyte subsets. In this paper we analyzed the UK patients for their immunological response to the Nap treatment in relation to their potential benefit and prolonged overall survival.

## RESULTS

### Patients

Eighteen of the 40 UK patients with RCC received Nap (15 μg/kg given intravenously in three cycles of four once-daily injections) plus IFN-α (9 MU subcutaneously three times weekly) and 22 UK patients received the same dose and schedule of IFN-α monotherapy except for weeks with Nap treatment. Patient characteristics are summarized in Table 1. Thirty two out of 40 patients received first line treatment. Twenty three patients had good and 17 patients had intermediate MSKCC risk score. The UK population was rather well balanced although a slight overrepresentation of patients with intermediate MSKCC risk score and higher IL-6 was seen in the IFN-α monotherapy arm.

**Table 1:** Demographic and baseline characteristics of the ITT and UK populations

		ITT Nap+IFN-α (*n* = 253)	IFN-α (*n* = 260)	UK Nap+IFN-α (*n* = 18)	IFN-α (*n* = 22)
Age, median (range)		58 (25–79)	57 (19–83)	64 (46–76)	61 (44–76)
Sex, n (%)	Females	70 (28)	77 (30)	4 (22)	9 (41)
	Males	183 (72)	183 (70)	14 (78)	13 (59)
Ethnic origin, n (%)	White	253 (100)	258 (99)	18 (100)	22 (100)
	Asian	0 (0)	2 (1)	0 (0)	0 (0)
ECOG performance status, n (%)	0	164 (65)	159 (61)	17 (94)	21 (95)
	1	89 (35)	100 (39)	1 (6)	1 (5)
MSKCC risk subgroup, n (%)	Good	152 (60)	152 (59)	11 (61)	12 (55)
	Intermediate	101 (40)	108 (42)	7 (39)	10 (45)
Line of treatment, n (%)	1st	248 (98)	250 (96)	15 (83)	17 (77)
	2nd	5 (2)	10 (4)	3 (17)	5 (23)
Histopathological type, n (%)	Clear cell carcinoma	237 (94)	238 (92)	14 (78)	17 (77)
	Papillary cell carcinoma	13 (5)	18 (7)	3 (17)	5 (23)
	Other	3 (1)	4 (2)	1 (6)	0 (0)
Baseline biomarkers, median	Anti-SEA/E-120 pmol/mL	53	54	34	29
	IL-6 pg/mL	6.7	7.2	4.5	9.3

### Changes of lymphocyte subsets in blood during Nap treatment

**Figure 1:**
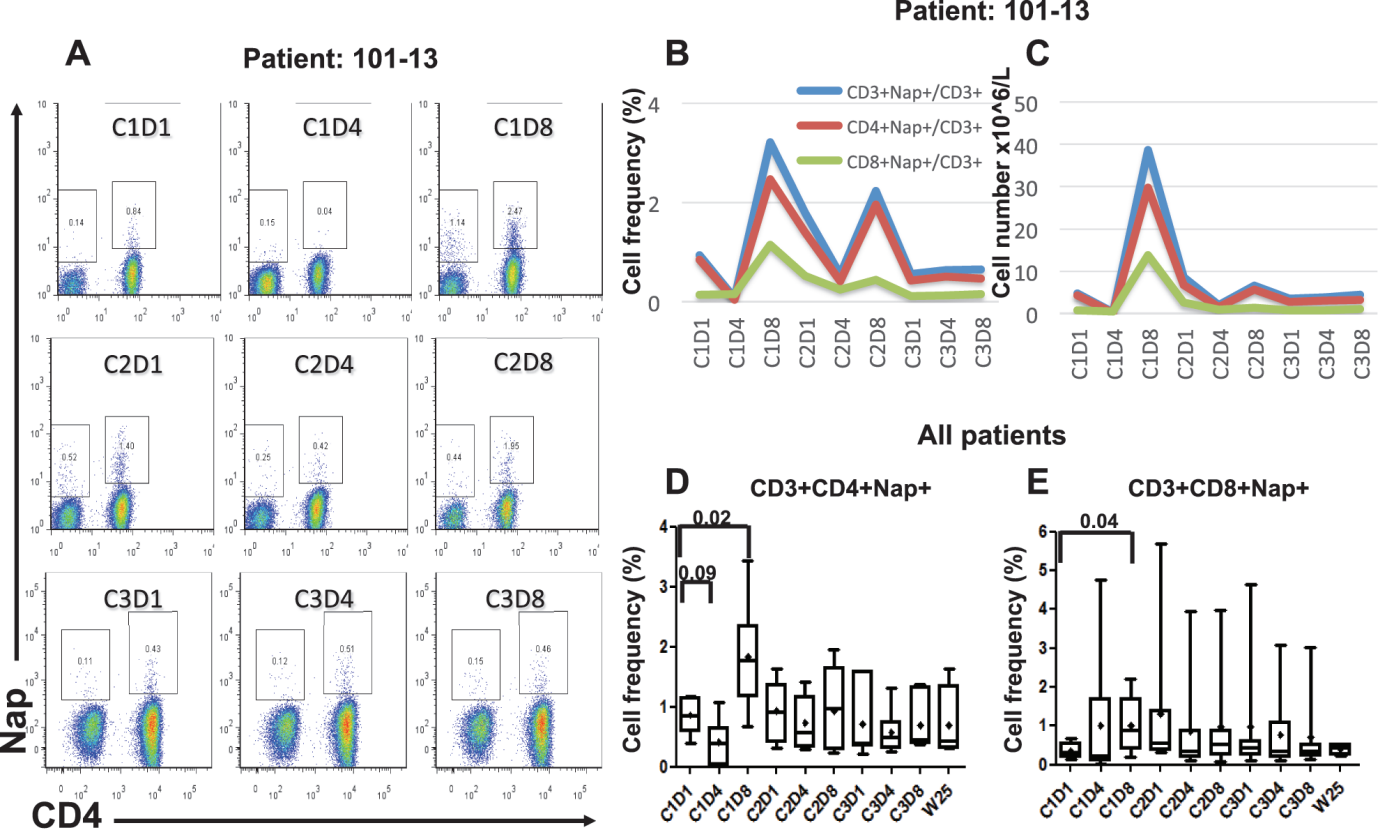
Flow cytometric and overall plots showing the percentage of Nap-specific CD4^+^ and CD8^+^ (CD3^+^CD4^−^) T cells in PBMCs of patients pre- and several time points after start of Nap treatment Cells were gated on lymphocytes and CD3^+^ T cells. Flow cytometric plots for patient 101–13 are shown in **(A)**. Absolute number of Nap-specific T cells was roughly estimated using the absolute numbers obtained from full blood counts. The overall percentages and absolute numbers of Nap-specific T cells for patient 101–13 are shown in **(B and C)**, respectively. Box and whiskers plots showing minimum, maximum, lower and upper quartiles and median of CD3^+^CD4^+^Nap^+^ T cells **(D)** and CD3^+^CD8^+^Nap^+^ T cells **(E)** for all patients before and during/following Nap treatment. Means are shown with diamonds and the forks depict *p*-values for differences between time points.

**Figure 2:**
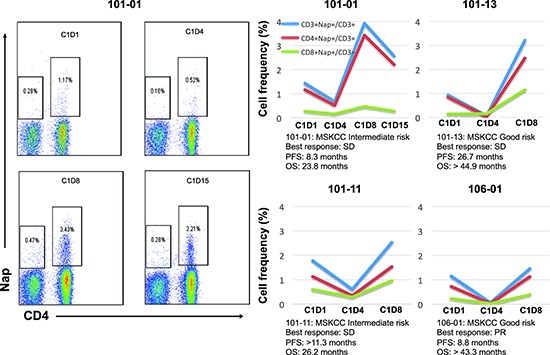
The overall percentages of Nap-specific T cells for the four patients with the most pronounced changes in these subsets are shown before and during/after the first cycle of treatment The flow cytometric plots show the levels of Nap-specific T cells of patient 101–01 before and three time points after treatment. Prognosis and clinical responses for these patients are depicted.

Nine patients donated blood for lymphocyte subset analysis during Nap treatment. Four-color flow cytometric assays were performed to measure the level of Nap-specific T cells in the peripheral blood of patients prior to and after treatment with Nap. T lymphocytes expressing T cell receptors containing TRBV7–9 bind Nap and can be activated by Nap [11]. An example of flow cytometric analysis during the three Nap treatment cycles is shown in Figure 1A. Levels of Nap-specific CD4^+^ and CD8^+^ T cells for all patients at all analyzed time points are shown in Figures 1D and 1E, respectively. At baseline, Nap-specific CD4^+^ T cell level (0.86%) was significantly higher than Nap-specific CD8^+^ T cells (0.35%) (*p* = 0.001). The frequency of Nap-specific CD4^+^ T cells in peripheral blood was reduced after 3 treatment days in samples collected pre-dose on day 4 (C1D4, *p* = 0.09). However, levels of both Nap-specific CD4^+^ and CD8^+^ T cells were significantly higher 8 days after the first treatment (C1D8, *p* = 0.02 and 0.04), as shown in Figure 1. The total number of T lymphocytes, Nap-specific as well as non-specific (data not shown), were reduced in peripheral blood after 3 days of treatment and expanded on day 8, 4 days after the last Nap injection. The four patients (patients 101–01, 101–11, 101–13 and 106–01) with the most pronounced Nap-specific T lymphocyte reduction on day 4 and expansion on day 8 are depicted in Figure 2. The expansion of Nap-specific T cells at C1D8 was higher within the CD4^+^ T-cell subpopulation, compared to the CD8^+^ T cell subpopulation. In addition, patient 101–13 showed increased Nap-specific T cell frequencies at day 8 of cycle 2 (C2D8) as well (Figure 1B).

**Figure 3:**
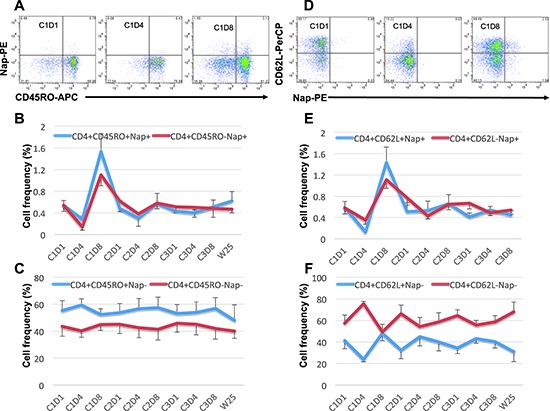
Expression of CD45RO and CD62L within CD4^+^Nap^+/−^ T cells Examples of flow cytometric plots in PBMCs of patient 101–13 pre and two time points during cycle 1 of treatment are shown in **(A and D)**. Cells were gated on lymphocyte and CD4^+^ T cells. The figures show the mean percentage +/− SEM of CD45RO^+/−^ within CD4^+^Nap^+^ **(B)** and CD4^+^Nap^−^ T cells **(C)**; and the mean percentage +/− SEM of CD62L^+/−^ within CD4^+^Nap^+^ **(E)** and CD4^+^Nap^−^ T cells **(F)** for all patients before and at all investigated time points during/following treatment.

Some patients (e.g. 101–13; Figures 1B and 1C) showed expansion of CD8^+^Nap^+^ T cells but in general Nap^+^ T cells were detected mainly (>70%) within the CD4^+^ T population and most patients showed an expansion of CD4^+^Nap^+^ T cells (Figures 1D and 1E). The CD4^+^Nap^+^ T cells were analyzed for different distinct subsets referring to memory (CD45RO), homing to central lymphoid organs (CD62L) and suppression (Foxp3). Figure 3A shows an example of the flow cytometric analysis, and CD45RO^+/−^ within CD4^+^Nap^+^ and CD4^+^Nap^−^ T cells for all analyzed patients are depicted in Figures 3B and 3C. Both CD45RO^+^ and CD45RO^−^ cells were reduced in CD4^+^Nap^+^ T cells at C1D4, but CD45RO expression was still higher (0.28% versus 0.14%, *p* = 0.06, Figure 3B). Interestingly, a majority of the expanded CD4^+^Nap^+^ T cells displayed a memory phenotype; CD45RO was expressed in 58% of CD4^+^Nap^+^ T cells at C1D8 (*p* = 0.16, Figure 3B). There were no significant changes in frequency of CD45RO^+^ cells within CD4^+^Nap^−^ T cells following treatment (Figure 3C).

Both CD62L^+^ and CD62L^−^ cells were reduced in CD4^+^Nap^+^ T cells at C1D4 (Figure 3D and 3E), but the percentage of CD62L^+^ cells was significantly lower (0.13% versus 0.35%, *p* = 0.02). Lack of CD62L expression at C1D4 could indicate that the majority of CD4^+^Nap^+^ T cells expressing CD62L had entered the secondary lymphoid tissues as CD62L is a receptor allowing lymphocytes to home to such lymphoid tissues. Interestingly, when CD4^+^Nap^+^ T cells were expanded in peripheral blood at C1D8, there was a tendency for more cells expressing CD62L (1.43% versus 1.11%, *p* = 0.25). For CD4^+^Nap^−^ T cells, again the majority of cells (76%, Figure 3F) lacked CD62L expression at C1D4 (*p* = 0.001), with no difference at C1D8 (*p* = 0.84, Figure 3F).

**Figure 4:**
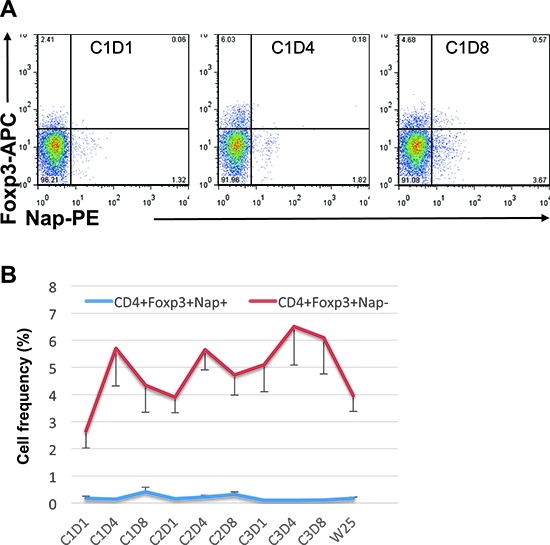
Foxp3 expression within CD4^+^Nap^+/−^ T cells Example of flow cytometric plots in PBMCs of patient 101–13 pre and two time points during cycle 1 of treatment is shown in **(A)**. Cells were gated on lymphocytes and CD4^+^ T cells. The figure shows the mean percentage +/− SEM of cells having Foxp3 expression within CD4^+^Nap^+^ and CD4^+^Nap^−^ T cells **(B)** for all patients before and at all investigated time points during/following treatment.

We investigated levels of Foxp3^+^ Treg cells in peripheral blood of patients before and after treatment. Patient 101–13 showed expansion of CD4^+^Foxp3^+^ Tregs during all cycles of treatment and they bounced to baseline level by the end of study (data not shown). Overall analysis for all patients showed the expansion of CD4^+^Foxp3^+^Nap^−^ Tregs; Treg levels were significantly higher at most time points after treatment, compared to pre-treatment level. Of note, the Treg expansion was detected mainly after 3 days of treatment within any of the cycles (Figure 4B). Immunotherapeutic modalities such as therapeutic cancer vaccines may expand tumour antigen-specific Tregs as well as antigen-specific T effector cells, which can induce immune suppression and dampen the expansion of tumour antigen-specific T effector cells [13]. Nap staining was combined with Foxp3 intracellular staining to determine the levels of Nap-specific Foxp3^+^ Tregs. Prior to treatment, Foxp3 was expressed mainly within CD4^+^Nap^−^ T cells, compared to CD4^+^Nap^+^ T cells (*p* = 0.006). Interestingly, Foxp3 expansion was seen within the CD4^+^Nap^−^ T cells, but not within the CD4^+^Nap^+^ subpopulation at any time point after treatment (Figures 4A and 4B).

### Cytokine production during Nap treatment

**Figure 5:**
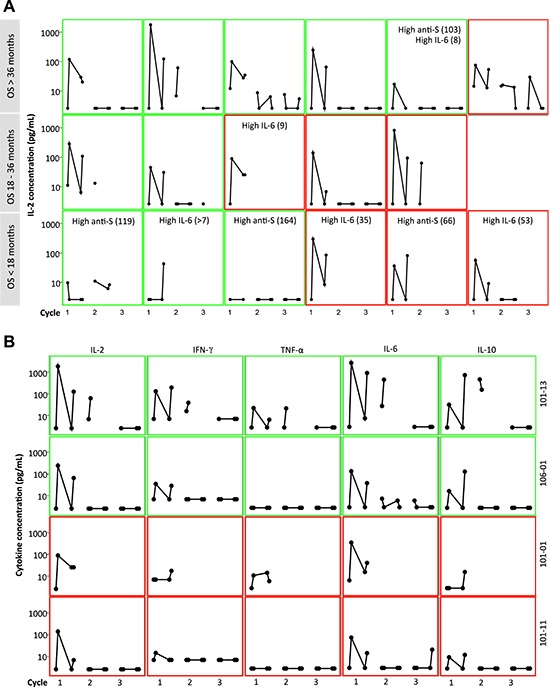
Cytokine response (pg/mL) in plasma at pre-dose and 3 hours after the first and second day of 3 cycles of Nap treatment. Green frame shows Good MSKCC risk score and red frame shows Intermediate MSKCC risk score **(A)**: IL-2 response in 17 of 18 patients from the UK. The patients were categorized according to their OS. Patients having over median of baseline anti-SEA/E-120 (High anti-S; > 53.5 pmol/mL) or IL-6 (High IL-6; > 7 pg/mL) are depicted. **(B)**: IL-2, IFN-γ, TNF-α, IL-6 and IL-10 and response in the four patients with the most pronounced Nap-specific T lymphocyte reduction on day 4 and expansion on day 8 (patients 101–01, 101–11, 101–13 and 106–01).

Seventeen of 18 Nap treated patients were analyzed for cytokine production as response to Nap. Cytokine production was measured pre-dose and 3 hours after Nap injection and the resulting increased plasma concentration served as a biomarker for Nap induced T lymphocyte activation and expansion. IL-2 may serve as a surrogate marker for T lymphocyte expansion. The T cell cytokines IL-2 and IFN-γ were elevated 3 hours after Nap (Figure 5 and Table 2). In addition to IL-2 and IFN-γ, Nap caused an increase of other cytokines including IL-6, IL-10 and TNF-α (Figure 5B). For most patients the induced systemic IL-2 levels were greatest during the first treatment cycle and were negligible during cycles 2 and 3. Patient 101–13 having a pronounced T cell response, including expansion of T cells also in cycle 2, showed increased plasma levels of IL-2 and other cytokines also in cycle 2 (Figure 5B). The different cytokines had distinct timely profiles. The four patients (patients 101–01, 101–11, 101–13 and 106–01) with the most pronounced Nap-specific T lymphocyte reduction on day 4 and expansion on day 8 showed high IL-2 production as measured in plasma during the first Nap treatment cycle (Figure 5B). Furthermore, IL-2 production was most pronounced in the patients with low anti-SEA/E-120 (as below). The inverse relationship between anti-SEA/E-120 antibody concentration in plasma and IL-2 production was also shown for the whole study (ASCO annual meeting 2013 abstract ID 3073, European Cancer Congress (ECCO) 2013 abstract ID 2710 and manuscript submitted for publication).

**Table 2:** IL-2 and IFN-γ response in plasma at 3 hours after the first and second day of Nap treatment and IL-6 and anti-SEA/E-120 at baseline in all patients from the UK, UK patients with OS < 18 months and UK patients with OS > 36 months. Geometric Mean (SE of Geometric Mean)

Biomarker	All (*n* = 15–17)	OS < 18 months (*n* = 5–6)	OS > 36 months (*n* = 5–6)	Spearman Correlation^*^ (*p*-value, *n* = 10–12)
IL-2 Day 1 3 h (pg/mL)	82 (38)	21.0 (24.3)	138 (96)	0.46 (0.15)
IL-2 Day 2 3 h (pg/mL)	34.7 (10.3)	23.4 (18.1)	50.2 (15.6)	0.17 (0.63)
IFN-γ Day 1 3 h (pg/mL)	19.2 (4.2)	14.7 (4.8)	26.7 (10.8)	0.35 (0.29)
IFN-γ Day 2 3 h (pg/mL)	19.6 (4.9)	13.0 (5.2)	27.7 (16.1)	0.22 (0.55)
IL-6 Baseline (pg/mL)	5.4 (1.1)	9.0 (5.2)	5.2 (1.3)	−0.37 (0.24)
Anti-SEA/E-120 Baseline (pmol/mL)	40.1 (6.3)	63.0 (18.4)	35.2 (8.7)	−0.48 (0.11)
IL-2 Day 1 3 h / IL-6 Baseline	16.7 (10.3)	1.65 (1.16)	44.6 (44.1)	0.75 (0.008)
IL-2 Day 2 3 h / IL-6 Baseline	7.3 (3.3)	1.84 (1.74)	19.8 (10.5)	0.66 (0.037)

* Spearman Correlation compares the Biomarker for patients with OS < 18 months and OS > 36 months, patients between 18 and 36 months are excluded.

### Antibodies binding to Nap (anti-SEA/E-120) and interleukin-6 at baseline

Nap is a fusion protein of bacterial and mouse origin. The superantigen moiety of Nap has been engineered to have low binding to baseline human antibodies [8]. Despite this fact, increased baseline anti-SEA/E-120 antibody levels were detected in certain territories predicting for suboptimal exposure, which may affect drug activity and anti-tumour efficacy (ASCO annual meeting 2013 abstract ID 3073, European Cancer Congress (ECCO) 2013 abstract ID 2710 and manuscript submitted for publication). Baseline levels of antibodies binding to SEA/E-120 were detectable in all patients and they had a median of 53.5 pmol/mL in the whole study. In the UK subset of patients 17 of 18 Nap treated patients and 14 of the control patients were analysed for anti-SEA/E-120 antibody levels at baseline. The median level of anti-SEA/E-120 in the UK patients was 34 pmol/mL, which is lower as compared to other study territories and at similar level as recorded in phase 1 [12].

The patients were also analysed for IL-6 in plasma at baseline. Baseline levels of IL-6 were detectable in most patients (22% < LLOQ) and they had a median of 7 pg/mL in the whole study, similar to the UK subset. At normal and low levels of IL-6, disease has not yet tipped the immune status into suppression. Accordingly patients with non-elevated systemic IL-6 levels would be expected to have the best chance of responding to immunotherapy. Indeed baseline plasma IL-6 was predictive of benefit with tumour vaccines [14, 15] but also for pazopanib [16].

Geometric means of anti-SEA/E-120 and IL-6 in the Nap treated UK subset of patients were 40.1 pmol/mL and 5.4 pg/mL, respectively (Table 2). Therefore this population had a favourable baseline profile for responding to the Nap immunotherapy as compared to the whole study population and the majority of treated UK patients analysed for the baseline biomarkers had low levels of the predictive biomarkers (Figure 5A).

### Overall survival in the UK patient subset with focus on patients with Nap induced T lymphocyte response

**Figure 6:**
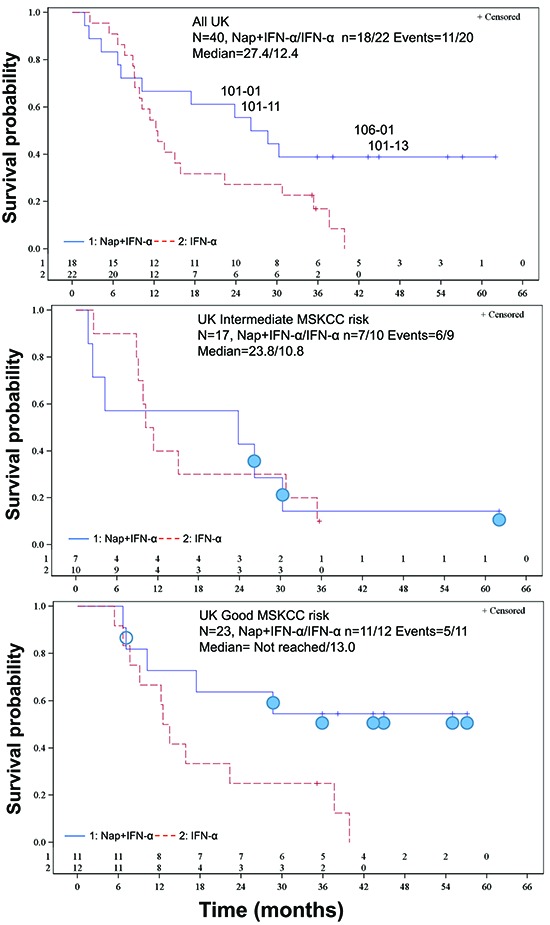
Kaplan-Meier OS plots for the UK patients **(A)** All UK-patients. HR (95% CI) = 0.56 (0.26, 1.19), *p* = 0.13 stratified for MSKCC risk score. The four patients with the most pronounced Nap-specific T lymphocyte reduction on day 4 and expansion on day 8 (patients 101–01, 101–11, 101–13 and 106–01) are depicted. **(B)** Intermediate MSKCC risk score patients. HR (95% CI) = 1.03 (0.36, 2.95), *p* = 0.95. Filled circle shows patient having below median of baseline anti-SEA/E-120 (<53.5 pmol/mL) and IL-6 (<7 pg/mL). **(C)** Good MSKCC risk score patients. HR (95% CI) = 0.32 (0.11, 0.93), *p* = 0.029. Filled circle shows patient having below median of baseline anti-SEA/E-120 (<53.5 pmol/mL) and IL-6 (<7 pg/mL). Open circle shows that baseline anti-SEA/E-120 and IL-6 is missing.

A total of 373 deaths (73% of patients) had occurred at the predefined final OS analysis in the ITT population. Median OS for the patients treated with Nap+IFN-α was 17.1 months versus 17.5 months for the patients receiving IFN-α alone. No difference of OS between treatment arms in the ITT population was detected and accordingly the primary endpoint OS was not reached for the whole phase 2/3 study, Hazard Ratio (HR) = 1.08. PFS showed HR of 0.92. Anti-SEA/E-120, a biomarker for drug exposure, and IL-6, a biomarker for immune responsiveness, were assessed as predictive baseline biomarkers. In a subgroup of patients having below median of baseline anti-SEA/E-120 and IL-6, prolonged PFS (HR = 0.62, *p* = 0.016) and OS (HR = 0.59, *p* = 0.020) were achieved (ASCO annual meeting 2013 abstract ID 3073, European Cancer Congress (ECCO) 2013 abstract ID 2710 and manuscript submitted for publication). 31 deaths (78% of patients) had occurred at the predefined final OS analysis in the UK population. Median OS for the UK patients treated with Nap+IFN-α was 27.4 months versus 12.4 months for the patients receiving IFN-α alone (Figure 6A; HR (95% CI) = 0.56 (0.26, 1.19), *p* = 0.13 stratified for MSKCC risk score).

The 17 UK patients analysed for IL-2 during Nap treatment were defined according to MSKCC risk (good or intermediate) and if they had overall survival below 18 months, between 18 and 36 months or more than 36 months. A clear tendency of higher IL-2 responses was seen in patients surviving more than 36 months (Table 2). All but one Nap treated patients surviving more than 18 months showed IL-2 response after treatment. The patient lacking IL-2 response (<20 pg/mL) with long survival had high baseline levels of anti-SEA/E-120. The patients surviving less than 18 months responded poorly with IL-2 and/or had high baseline levels of anti-SEA/E-120 or IL-6. The four patients (patients 101–01, 101–11, 101–13 and 106–01) with the most pronounced Nap-specific T lymphocyte reduction on day 4 and expansion on day 8 showed high IL-2 production as measured in plasma during the first Nap treatment cycle (Figure 2 and Figure 5B) and survived longer than 18 months (Figure 6). Patients 101–01 and 101–11 were MSKCC intermediate risk patients with survival longer than 23 months and patients 101–13 and 106–01 (106–01 showing PR) were MSKCC good risk patients with long survival (> 40 months) (Figures 6B and 6C). Interestingly, patients with OS > 36 months showed significantly (*p* < 0.05) higher ratio of plasma IL-2 level at 3 hours after the first and second day of Nap treatment to IL-6 baseline level, compared to patients with OS < 18 months (Table 2).

Both intermediate and good MSKCC risk patients with low levels of baseline anti-SEA/E-120 and IL-6 responding with IL-2 in blood plasma and blood T cell reduction day 4 and expansion day 8 after start of the Nap treatment cycle were found to be among patients with relatively long survival indicating that this biomarker pattern might be important for clinical activity.

## DISCUSSION

Although the phase 2/3 study did not meet its primary endpoint, addition of Nap to IFN-α improved OS (HR: 0.59; *p* = 0.020) and PFS (HR: 0.62; *p* = 0.016) in a subgroup of patients with normal levels of anti-SEA/E-120 antibodies and low IL-6 levels. In this paper we have analysed the Nap induced immune response in a cohort of the UK patients to characterize baseline biomarkers and immune response patterns in relation to potential benefit from Nap treatment.

The superantigen moiety of Nap has been engineered to have low binding to baseline human antibodies [8]. Despite this fact increased anti-SEA/E-120 antibody levels may affect drug activity and anti-tumour efficacy. In the phase 2/3 study the baseline concentration of anti-SEA/E-120 antibodies was unexpectedly higher in certain territories and exposure accordingly lower. Most patients in the phase 1 studies, all having low anti-SEA/E-120 antibody titres, were from the US, the UK and Scandinavia and as expected most patients in this study from the UK had also low concentrations of baseline anti-SEA/E-120.

IL-6 is implicated in the pathophysiology of various solid tumours. High IL-6 levels are prognostic and correlate with tumour metastasis, disease stage, and short survival in several cancers including RCC [17–19]. The IL-6/Janus kinase (JAK)/Signal transducer and activator of transcription (STAT) 3 pathway is one of the most important signaling pathways associated with tumour development and induction of tumour induced immune suppression [20]. IL-6 is therefore a biomarker for immune status of the tumour microenvironment and for a patient's chronic inflammation/immune suppression status in general. At normal and low levels of IL-6, disease has not yet tipped the immune status into suppression. Accordingly patients with non-elevated systemic IL-6 levels would be expected to have the best chance of responding to immunotherapy. Indeed baseline plasma IL-6 was predictive of benefit with tumour vaccines [14, 15] but also for pazopanib [16]. In the phase 2/3 study IL-6 was shown to be both prognostic as well as predictive for Nap activity since the subgroup and a trend analysis clearly supported that low baseline anti-SEA/E-120 and IL-6 plasma levels independently predict anti-tumour efficacy after Nap+IFN-α treatment.

Generally in the patients from the UK cohort, the expansion of Nap^+^ T cells was detected in peripheral blood during first cycle of treatment, usually at day 8. The baseline biomarkers for Nap exposure and/or low tumour induced immune suppression coincided with IL-2 production after Nap treatment as well as the expansion of Nap^+^ T cells. Interestingly, the expansion of Nap-specific CD4^+^ T cells was detected in Foxp3^−^ and not Foxp3^+^ cells, confirming that Nap expands Nap-specific T effector cells but not Nap-specific Tregs.

The subset of patients with low levels of antibodies towards Nap and low IL-6, indicating that tumour induced immune suppression has not yet been established, and having relatively early disease (i.e. good MSKCC risk score), seems to have the best benefit, as evaluated by OS, of the Nap immunotherapy. The immune cells of this subset of patients can be activated to pronounced IL-2 production resulting Nap-specific expansion of CD4^+^ and CD8^+^ T cells which probably is a prerequisite for optimal Nap induced anti-tumour effects. In previous studies we have shown that T cells infiltrate tumours after Nap treatment in responding patients [12]. Therefore, it may be speculated that Nap acts in two phases; 1) Induction of T cell activation, proliferation and maturation into effector cells, 2) Recruitment to and T cell killing (SADCC) of the tumour.

IL-2 may serve as a surrogate marker for T cell expansion. Hypothetically, Nap treatment induced anti-tumour effects require T cell activation and expansion in advance to that the T effectors infiltrate the tumour to cause tumour cell elimination. The patients in the UK subset of this phase 2/3 study showed a tendency of OS benefit after Nap treatment. Most Nap treated patients with long OS had low baseline plasma IL-6 and normal levels of anti-SEA/E-120. Furthermore, patients with pronounced Nap induced IL-2 in plasma and verified T cell expansion on day 8 after initiation of Nap treatment had long OS. In conclusion, patients with low baseline IL-6 and anti-SEA/E-120 may respond well to Nap by triggering T cell activation and expansion (IL-2) paving the way for anti-tumour effects documented as prolonged OS. Being a very efficient T cell trigger and stimulator of tumour infiltrating T cells, Nap may also represent an optimal combo to checkpoint immunotherapy such as anti-PD-1 therapy [21].

## MATERIALS AND METHODS

### Patients

From May 2007 to October 2010, 513 RCC patients in Bulgaria (76), Romania (56), Russia (188), Ukraine (153) and UK (40) were randomized 1:1 and treated with Nap+IFN-α or IFN-α in a multinational, multicenter, randomized, open-label, parallel-group, phase 2/3 study in patients with confirmed metastatic or inoperable locally advanced RCC eligible for standard therapy with IFN-α. Results from the whole study will be published elsewhere. Additional key eligibility criteria were histologically or cytologically confirmed clear cell or papillary type RCC, Karnofsky performance status ≥ 70, good or intermediate risk group prognosis by MSKCC risk score criteria (score 0–2), life expectancy > 3 months and acceptable levels of specific haematology and serum chemistry parameters. The patients from the UK were selected for additional analysis of immune response during Nap treatment in relation to their potential benefit from Nap as evaluated by overall survival.

### Study design

After screening and enrolment, patients with RCC were randomized 1:1 to receive Nap+IFN-α or established treatment with IFN-α. Stratification was to establish balance between the treatment arms with regard to a prognostic index based on MSKCC risk and first versus second line of treatments. Patients in the active arm 1 were given 15 μg/kg Nap intravenously in three cycles of four once daily injections plus IFN-α, 9 MU subcutaneously three times weekly, except for the Nap treatment weeks or the same dose and schedule of IFN-α monotherapy. Nap treatment cycles were given weeks 1, 9 and 17 and IFN-α during all other weeks up to 18 months and IFN-α monotherapy in treatment arm 2 was given from week 1 up to 18 months. The primary endpoint was overall survival (OS). Secondary endpoints were PFS, response rate, immunological response to treatment in patients receiving Nap and safety. The main analysis on OS data from all patients was predefined to be executed at expected 383 events.

### Biomarkers and immunopharmacology

Multiple blood samples were collected at specific time points (baseline and during treatment) for anti-SEA/E-120 and cytokines. Cytokines (IL-2, IL-6, IL-10, IFN-γ and TNF-α) as a measure for immune activation were assayed at 0 and 3 hours on Days 1 and 2 of the Nap treatment cycles. Baseline plasma IL-6 and all anti-SEA/E-120 concentrations were measured with ELISA methods (Quantikine, R&D Systems and modified from [12]) and the plasma cytokine response patterns were analysed with a cytometric bead array (BD Biosciences).

Forty ml blood samples were collected in heparinized tubes from nine patients with extended consents for lymphocyte phenotype analyses. Blood samples were collected pre-dose, at dosing Day 1 and Day 4 of Weeks 1, 9 and 17 (Day 1, Day 4, Day 57, Day 60, Day 113 and Day 116 respectively), at Day 8, at Day 64, at Day 120 and at week 25, which represent cycle 1 day 1 (C1D1, baseline), C1D4, C1D8, C2D1, C2D4, C2D8, C3D1, C3D4, C3D8 and week 25.

Peripheral blood mononuclear cells (PBMCs) were separated by Ficoll Hypaque density centrifugation and cells were collected from the interface, washed twice in PBS then frozen at a concentration of 1 × 10^7^/ml in freezing media (90% FCS and 10% DMSO) and stored in Liquid Nitrogen until required. A reagent, the Nap/strep-PE complex, was used to detect SEA/E-120 binding T cells [11]. The Nap/strep-PE complex was used in combination with CD3-PerCP, CD4-FITC and CD8-APC antibodies to measure the percentage of CD3^+^CD4^+^ and CD3^+^CD8^+^ T cells specific for SEA/E-120. CD62L and CD45RO were used to measure the frequency of naïve and memory Nap^+^ and Nap^−^ cells within CD4^+^ and CD8^+^ T cells before and after treatment. Frozen PBMCs were thawed and stained with Nap/strep-PE conjugate in addition to different monoclonal antibodies before flow cytometric analysis using FACSCalibur or FACSCanto II (BD Biosciences) flow cytometers. Data analysis was performed using FlowJo version 7.5.5 software (TreeStar, Ashland, OR, USA). T regulatory cells (Tregs) were determined by their expression of CD4 and Foxp3. For detection of Nap^+^ and Nap^−^ cells within CD4^+^Foxp3^+^ Tregs, cells were first stained for extracellular CD4 and Nap markers using anti-human CD4-PerCP and anti-human Nap/strep-PE. Following fixation and permeabilization using eBioscience fix/perm buffers, the cells were washed and blocked for non-specific binding sites using normal rat serum. Then anti-human Foxp3-APC or rat IgG2a-APC isotype negative control (eBioscience) were added for 30 minutes before washing twice and flow cytometric analysis.

### Statistical methods and analysis

The statistics in this paper should be seen as descriptive as the sample size in this sub-study is not high enough to detect differences in OS. Furthermore, statistical tests are presented and no adjustment for multiplicity has been done. Testing of OS versus treatment arms was done with the log-rank test and the hazard ratio with 95% confidence intervals was calculated with the Cox Regression methods, both stratified for MSKCC risk score (SAS, version 9.3, PROC LIFETEST and PHREG). Tests of biomarkers were done with Student's paired and un-paired t-test. Test of Biomarker level for patients with OS with short versus long survival was done with Spearman's Correlation. Two-sided tests with *p*-values < 0.05 were regarded as significant.

## References

[1] Abe H, Kamai T (2013). Recent advances in the treatment of metastatic renal cell carcinoma. Int J Urol.

[2] Eisen T, Hedlund G, Forsberg G, Hawkins R (2014). Naptumomab estafenatox: targeted immunotherapy with a novel immunotoxin. Curr Oncol Rep.

[3] Dohlsten M, Abrahmsén L, Björk P, Lando PA, Hedlund G, Forsberg G, Brodin T, Gascoigne NR, Förberg C, Lind P, Kalland T (1994). Monoclonal antibody-superantigen fusion proteins: tumor-specific agents for T-cell-based tumor therapy. Proc Natl Acad Sci U S A.

[4] Dohlsten M, Hedlund G, Akerblom E, Lando PA, Kalland T (1991). Monoclonal antibody-targeted superantigens: a different class of anti-tumor agents. Proc Natl Acad Sci U S A.

[5] Dohlsten M, Hansson J, Ohlsson L, Litton M, Kalland T (1995). Antibody-targeted superantigens are potent inducers of tumor-infiltrating T lymphocytes *in vivo*. Proc Natl Acad Sci U S A.

[6] Cheng JD, Babb JS, Langer C, Aamdal S, Robert F, Engelhardt LR, Fernberg O, Schiller J, Forsberg G, Alpaugh RK, Weiner LM, Rogatko A (2004). Individualized patient dosing in phase I clinical trials: the role of escalation with overdose control in PNU-214936. J Clin Oncol.

[7] Shaw DM, Connolly NB, Patel PM, Kilany S, Hedlund G, Nordle O, Forsberg G, Zweit J, Stern PL, Hawkins RE (2007). A phase II study of a 5T4 oncofoetal antigen tumour-targeted superantigen (ABR-214936) therapy in patients with advanced renal cell carcinoma. Br J Cancer.

[8] Forsberg G, Skartved NJ, Wallén-Ohman M, Nyhlén HC, Behm K, Hedlund G, Nederman T (2010). Naptumomab estafenatox, an engineered antibody-superantigen fusion protein with low toxicity and reduced antigenicity. J Immunother.

[9] Griffiths RW, Gilham DE, Dangoor A, Ramani V, Clarke NW, Stern PL, Hawkins RE (2005). Expression of the 5T4 oncofoetal antigen in renal cell carcinoma: a potential target for T-cell-based immunotherapy. Br J Cancer.

[10] Elkord E, Shablak A, Stern PL, Hawkins RE (2009). 5T4 as a target for immunotherapy in renal cell carcinoma. Expert Rev Anticancer Ther.

[11] Hedlund G, Eriksson H, Sundstedt A, Forsberg G, Jakobsen BK, Pumphrey N, Rödström K, Lindkvist-Petersson K, Björk P (2013). The tumor targeted superantigen ABR-217620 selectively engages TRBV7–9 and exploits TCR-pMHC affinity mimicry in mediating T cell cytotoxicity. PloS one.

[12] Borghaei H, Alpaugh K, Hedlund G, Forsberg G, Langer C, Rogatko A, Hawkins R, Dueland S, Lassen U, Cohen RB (2009). Phase I dose escalation, pharmacokinetic and pharmacodynamic study of naptumomab estafenatox alone in patients with advanced cancer and with docetaxel in patients with advanced non-small-cell lung cancer. J Clin Oncol.

[13] Zhou G, Drake CG, Levitsky HI (2006). Amplification of tumor-specific regulatory T cells following therapeutic cancer vaccines. Blood.

[14] Harrop R, Treasure P, de Belin J, Kelleher M, Bolton G, Naylor S, Shingler WH (2012). Analysis of pre-treatment markers predictive of treatment benefit for the therapeutic cancer vaccine MVA-5T4 (TroVax). Cancer Immunol Immunother.

[15] Quoix E, Ramlau R, Westeel V, Papai Z, Madroszyk A, Riviere A, Koralewski P, Breton JL, Stoelben E, Braun D, Debieuvre D, Lena H, Buyse M (2011). Therapeutic vaccination with TG4010 and first-line chemotherapy in advanced non-small-cell lung cancer: a controlled phase 2B trial. Lancet Oncol.

[16] Tran HT, Liu Y, Zurita AJ, Lin Y, Baker-Neblett KL, Martin AM, Figlin RA, Hutson TE, Sternberg CN, Amado RG, Pandite LN, Heymach JV (2012). Prognostic or predictive plasma cytokines and angiogenic factors for patients treated with pazopanib for metastatic renal-cell cancer: a retrospective analysis of phase 2 and phase 3 trials. Lancet Oncol.

[17] Blay JY, Negrier S, Combaret V, Attali S, Goillot E, Merrouche Y, Mercatello A, Ravault A, Tourani JM, Moskovtchenko JF, Philip T, Favrot M (1992). Serum level of interleukin 6 as a prognosis factor in metastatic renal cell carcinoma. Cancer Res.

[18] Negrier S, Perol D, Menetrier-Caux C, Escudier B, Pallardy M, Ravaud A, Douillard JY, Chevreau C, Lasset C, Blay JY (2004). Interleukin-6, interleukin-10, and vascular endothelial growth factor in metastatic renal cell carcinoma: prognostic value of interleukin-6—from the Groupe Francais d'Immunotherapie. J Clin Oncol.

[19] Thiounn N, Pages F, Flam T, Tartour E, Mosseri V, Zerbib M, Beuzeboc P, Deneux L, Fridman WH, Debré B (1997). IL-6 is a survival prognostic factor in renal cell carcinoma. Immunol Lett.

[20] Yu H, Pardoll D, Jove R (2009). STATs in cancer inflammation and immunity: a leading role for STAT3. Nat Rev Cancer.

[21] Kim HJ, Cantor H (2014). The Path to Reactivation of Antitumor Immunity and Checkpoint Immunotherapy. Cancer Immunol Res.

